# Lupin as a Source of Bioactive Antioxidant Compounds for Food Products

**DOI:** 10.3390/molecules28227529

**Published:** 2023-11-10

**Authors:** Lorenzo Estivi, Andrea Brandolini, Andrea Gasparini, Alyssa Hidalgo

**Affiliations:** 1 Department of Food, Environmental and Nutritional Sciences (DeFENS), University of Milan, Via Celoria 2, 20133 Milan, Italy; lorenzo.estivi@unimi.it (L.E.); alyssa.hidalgovidal@unimi.it (A.H.); 2 Council for Agricultural Research and Economics, Research Centre for Animal Production and Aquaculture (CREA-ZA), Via Piacenza 29, 26900 Lodi, Italy; andrea.gasparini@crea.gov.it

**Keywords:** carotenoids, flavonoids, *Lupinus*, phenolic acids, tocopherols

## Abstract

Four species of lupin (white lupin, yellow lupin, blue lupin and Andean lupin) are widely cropped thanks to the excellent nutritional composition of their seeds: high protein content (28–48 g/100 g); good lipid content (4.6–13.5 g/100 g, but up to 20.0 g/100 g in Andean lupin), especially unsaturated triacylglycerols; and richness in antioxidant compounds like carotenoids, tocols and phenolics. Particularly relevant is the amount of free phenolics, highly bioaccessible in the small intestine. However, the typical bitter and toxic alkaloids must be eliminated before lupin consumption, hindering its diffusion and affecting its nutritional value. This review summarises the results of recent research in lupin composition for the above-mentioned three classes of antioxidant compounds, both in non-debittered and debittered seeds. Additionally, the influence of technological processes to further increase their nutritional value as well as the effects of food manufacturing on antioxidant content were scrutinised. Lupin has been demonstrated to be an outstanding raw material source, superior to most crops and suitable for manufacturing foods with good antioxidant and nutritional properties. The bioaccessibility of lupin antioxidants after digestion of ready-to-eat products still emerges as a dearth in current research.

## 1. Introduction

Lupins are annual plants belonging to the Fabaceae family, genus *Lupinus*. More than 400 species are known [1], originating from the Mediterranean region, North Africa and North and South America. Four *Lupinus* species, i.e., white lupin (*Lupinus albus* L.), yellow lupin (*Lupinus luteus* L.), blue or narrow leaf lupin (*Lupinus angustifolius* L.) and Andean lupin (*Lupinus mutabilis* Sweet.) are widely grown as green manure, forage and food. *L. angustifolius*, *L. albus* and *L. luteus* (all from the Mediterranean region) are the most widespread species worldwide, while *L. mutabilis* (a.k.a. chocho or tarwi) is cultivated mainly in South America. According to the Food and Agriculture Organization (FAO), in 2021 the lupin cropped area was almost 1 million hectares worldwide, for a total production of 1.385 million metric tons (https://www.fao.org/faostat; last accessed on 23 September 2023); Australia is by far the most major lupin producer, with 865,619 t (about 63% of world production), followed by Poland (221,390 t), the Russian Federation (69,723 t), Morocco (56,856 t), Germany (53,400 t), Chile (37,049 t), Greece (15,830 t), Peru (15,790 t), France (15,130 t) and South Africa (9876 t) (https://www.fao.org/faostat; last accessed on 23 September 2023).

Lupins are considered improver crops because they can grow in a wide range of soil and climate conditions where other crops fail and because (like other legumes) they fix the atmospheric nitrogen in the soil through a symbiotic relationship with *Rhizobium* bacteria: the fixed nitrogen becomes available also for subsequent crops.

The fruit of the lupins is a pod that splits open along two seams. The seeds may be eaten as snacks, in salads or side dishes, while their flour is used in the preparation of many foods, like vegetable milk, cheese and meat, fermented products, mayonnaise, chips, noodles, pasta, baked goods, etc. [2,3,4,5,6,7,8]. Furthermore, from the seeds, it is possible to extract an oil rich in monounsaturated and polyunsaturated fatty acids [9,10] as well as protein isolates with outstanding physical, technological and functional properties [11,12,13]. The flour does not contain gluten-forming proteins and is suitable for the manufacturing of gluten-free foods for people with coeliac disease.

The aim of this review is to describe the results of current research on the content of carotenoids, tocols and phenolic compounds both in non-debittered and debittered seeds, as well as to provide some information on their fate during food processing.

## 2. Chemical Composition of the Seeds

The excellent composition of lupin seeds and flour confers to lupin-based foods many health benefits [14], thanks to anticancer, antimicrobial, antidiabetic, antihypertensive, antioxidant, anti-inflammatory and antimicrobial activities [15,16].

Lupin seeds are characterised by high protein content, which may vary from 28 to 48 g/100 g depending on species, variety, growing conditions and soil types [17,18]; the lowest values are found in *L. angustifolius* and the highest values in *L. mutabilis* [19,20,21,22]. The main storage proteins are globulins (80–90%), which are classified into four groups: α-conglutin (11S globulin), β-conglutin (7S globulin), γ-conglutin (7S basic globulin) and δ-conglutin (2S sulphur-rich albumin) [23,24]; prolamins and glutelins are scarce [25]. Lysine, isoleucine, leucine, phenylalanine and tyrosine are in proportions appropriate for adults [26], while methionine and cysteine (sulphur-containing amino acids), valine and tryptophan are the principal limiting compounds [27,28].

Lupin seeds contain high levels of dietary fibre [22,29], both before and after the removal of the hulls (a common step before milling). In fact, the flour on average contains 41.5 g/100 g dietary fibre (about 75% insoluble), which mainly consists of nonstarch polysaccharides (cellulose 79%, hemicellulose 14% and lignin 7%) located in the endosperm cell walls [30].

Lipid content in *L. albus*, *L. luteus*, and *L. angustifolius* seeds ranges from 4.6 to 13.5 g/100 g [18,31,32,33], while the *L. mutabilis* seeds have a higher concentration: Ruiz-Lopez et al. [18] determined a 14.7 g/100 g lipid content, while Briceño Berru et al. [19] and Carvajal-Larenas et al. [34] recorded even higher values (on average 16.1 and 18.9 g/100 g, respectively), but some genotypes even reached 20 g/100 g [35]. The oil extracted from lupin beans is characterised by scarce saturated fatty acids (10–27%) and abundant unsaturated fatty acids (78–87%), mainly oleic acid (25–63%), linoleic acid (13–57%) and linolenic acid (3–11%) [22,31,33,36,37,38], giving lupin oil outstanding nutritional and technological properties, while nonetheless maintaining remarkably high oxidative stability [9,39].

Lupin seeds contain very little starch (5–12%), while nonstarch polysaccharides are abundant. Cellulose, hemicellulose and pectin are found in the hulls, while the oligosaccharides are predominant in the cotyledons [23]. The total content of seed oligosaccharides (mainly stachyose and raffinose) varies depending on species, cultivar and environment: for example, in *L. albus* is 53.0 g/kg dry matter (DM) [40] while in *L. mutabilis* is 41.5 g/kg DM [41].

Lupin seeds are also a rich source of macro- (Ca, K, Na, and Mg) and microelements (Fe, Zn and Mn) [42,43], vitamins (thiamine, niacin and riboflain) and phytochemicals with antioxidant capacity such as carotenoids, tocopherols and phenolic compounds [19,41,44].

## 3. Antinutritional Factors: Quinolizidine Alkaloids

Compared to other legumes, lupin seeds are low in antinutritional factors such as trypsin inhibitors, lectines and saponins [29]. The content of phytic acid, a compound that reduces the bioavailability of some minerals through cation chelation, is very low (0.25–0.62 g/100 g in white lupin seeds) [29,45] and significantly inferior to soybean (1.54–2.27%) [45].

The main antinutritional compounds in lupin seeds are the quinolizidine alkaloids [27,46,47], which have a bitter taste and are often toxic, so before consumption, they must be removed through appropriate processes. The varieties with genetically low alkaloid content [48,49] have low yields, are more susceptible to pests and diseases and over time are inclined to regain their bitterness [49].

In general, debittering is achieved by removing the alkaloids from whole seeds using water as a solvent. Nevertheless, the chemical composition of the seeds is altered because several water-soluble molecules are also carried away: minerals, carbohydrates and oligosaccharides decrease while lipids and proteins increase as a consequence of concentration due to the elimination of the other compounds [20,50,51].

The traditional debittering method starts with preliminary hydration in water, followed by cooking and repeated washings for several days [50,52]. Besides disrupting cell walls and facilitating alkaloid removal, cooking blocks germination, coagulates proteins, inactivates enzymes and sanitises the product [53,54]. To shorten processing time and save water, several improved debittering methods have been proposed. Generally, they include longer boiling times [47], post-cooking washing with warm water [55], different rinsing solutions [47,56,57], germination [56,58], fermentation [59] or a combination of these methods [50,60]. Recently, Estivi et al. [61] reported that the use of sodium chloride or citric acid solutions significantly shortened debittering time, reduced water consumption and decreased alkaloid content to commercial values.

## 4. Carotenoids

The carotenoids, lipid-soluble antioxidants synthesised by many photosynthetic organisms, are classified as carotenes (tetraterpenoid hydrocarbons) or xanthophylls (with one or more oxygenated functions) [62]. Their single- and double-bond repeats influence the antioxidant properties, while the polar groups shape the interactions with the cellular membranes [63]. In the chloroplasts, the carotenoids act as light collectors and also safeguard against photosensitisation. The animals do not synthesise them, thus they are obtained from foods. The carotenoids are implicated in many human functions. For example, α- and β-carotenes are precursors of vitamin A, which is essential for proper visual functions, cellular reproduction and normal embryo development [64]. Furthermore, the carotenoids shield the cells from free radicals and singlet oxygen, avoiding oxidative damage. Zeaxanthin and lutein protect the macula region of the retina, contributing to the prevention of cataracts. Additional benefits linked to carotenoids are to protect against solar radiation, increase the immune response, hinder some types of cancer and assist in the prevention of cardiovascular and degenerative diseases [62,65].

Carotenoids (mainly lutein, but also α- and β-carotene, β-cryptoxanthin, zeaxanthin and violaxanthin) are scarce in lupins and therefore have not been extensively studied. Fernández-Marín et al. [66] observed 4.1 mg/kg DM in bitter seeds of *L. luteus*, while El-Difrawi and Hudson [67] reported 4.7 mg/kg in *L. albus*. In bitter seeds of *L. albus*, *L. luteus*, *L. angustifolius* and *L. mutabilis*, the carotenoid contents were 1.90–5.26, 0.99, 6.49 and 0.69–2.89 mg/kg DM, respectively [19]. Recently, Siger et al. [68] analysed 55 *L. angustifolius* breeding lines and observed values ranging from 3.02 to 5.65 mg/100 g; similarly, Siger et al. [31] found 8.9–12.7, 6.1–6.3 and 17.2–62.5 mg/kg in sweet *L. albus*, *L. luteus* and *L. angustifolius* genotypes, while Estivi et al. [69] observed a total carotenoid content ranging from 1.70 to 1.81 mg/kg in *L. mutabilis*. Interestingly, debittering tends to increase their concentration, due to the loss of several water-soluble molecules [19,44].

Hence, in *L. albus*, Estivi et al. [70] observed values ranging from 12.2 to 25.1 mg/kg DM, also as a function of the debittering solvent (Table 1).

## 5. Tocols

The tocols are another group of lipid-soluble antioxidants produced by photosynthetic organisms. They are separated into tocopherols (saturated phytyl group) and tocotrienols (tri-unsaturated phytyl group) but each class comprises four homologues (α, β, γ and δ), which differ in number and position of methyl groups in the chromane ring. For their capacity to reduce free radicals, in plants, they protect the photosynthetic apparatus from lipid peroxidation [71] and shield the polyunsaturated fatty acids from oxidation [72]. Like the carotenoids, the tocols are not synthesised by animals and must be acquired with food. Thanks to the capacity to quench the free radicals, they protect the polyunsaturated fatty acids in the membrane and limit free-radical damage in the tissues, contributing to the prevention of chronic diseases like cardiovascular, neurological and inflammatory diseases, cancer, etc. [73]. Only α-tocopherol has proven vitamin E activity [74] but the other tocols show comparable or even better antioxidant activity [75,76]; interestingly, the tocotrienols may even be superior to tocopherols in the prevention of cardiovascular disease [77].

As reported in Table 2, the most abundant tocol in lupins is γ-tocopherol (γ-T), representing > 97% of total tocols [19,31,32,78], followed by β-tocopherol (β-T) and δ-tocopherol (δ-T) (approximately 1% each) and by α-tocopherol (α-T; 0.3%). Briceño Berru et al. [19] reported that γ-tocopherol was plentiful in *L. luteus* and *L. mutabilis* and was less abundant in *L. angustifolius* and *L. albus*. Nevertheless, Siger et al. [31] and Martinez-Villaluenga et al. [78] observed higher γ-tocopherol concentrations in white lupin (126–130 and 201–516 mg/kg, respectively) than in yellow lupin (86–87 and 96–112 mg/kg). The total tocol content (Table 2) of *L. albus* can vary between 63 and 154 mg/kg [19,31,32,79] with a reported maximum of 581 mg/kg [78]; that of *L. luteus* may range from 86 to 187 mg/kg [19,31,78]; that of *L. angustifolius* can span between 65 and 123 mg/kg [19,31,32,68]; and that of *L. mutabilis* can vary from 169 to 250 mg/kg [19,41,44,69].

As described for the carotenoids, the debittering process can lead to an increase in the lipophilic and water-insoluble tocopherol content [19,70] due to the loss of several water-soluble compounds.

## 6. Free Phenolics

Another class of excellent oxygen radical scavengers includes the phenolic compounds, built by one or more aromatic rings with one or more hydroxyl groups. They are secondary metabolites produced by plants to counter environmental stresses that induce oxidative damage: the accrued synthesis of phenolic metabolites [80] stabilises the free radicals and defends membranes, lipids, proteins and DNA from oxidative harm [81]. Consequently, they safeguard the health of cell walls, providing anti-inflammatory, cardio-protective, antimicrobial, antithrombotic and vasodilatory effects [82]. The phenolics are found in three forms: soluble free, soluble conjugated, i.e., o-glycosylated to sugar moieties or other low molecular weight components, and insoluble bound, i.e., linked to cell wall constituents such as polysaccharides, protein, lignin, cutin or suberin [83]. In lupin, the most abundant fraction is by far the soluble free (>90%) [44], followed by the soluble conjugated and the insoluble bound (Table 3, Table 4, Table 5, Table 6 and Table 7).

Their bioavailability is related to their structural diversity because while the simplest free phenolic acids, like caffeic acid, can easily cross the intestinal barrier, more complex molecules are hardly absorbed [84]; nevertheless, after a bacteria-mediated partial degradation in the colon, they exhibit antioxidant capacity and may still perform a critical role in reducing the risk of bowel cancer [85].

**Table 3:** Free phenolics (mg/kg DM) of bitter (bit) and debittered (deb) seeds of *L. albus* with different solvents (* H_2_O; ** 1%NaCl; *** 1% citric acid).

Reference	[86]	[87]	[88]	[89]	[21]	[90]	[91]	[92]	[70]	[70]	[70]	[93]
	bit	bit	bit	bit	bit	bit	bit	bit	deb *	deb **	deb ***	deb *
	*n* = 11, 3 y	*n* = 1	*n* = 2	*n* = 11	*n* = 2	*n* = 4	*n* = 4	*n* = 2	*n* = 2	*n* = 2	*n* = 2	*n* = 3
**Total flavonoids**		460	0.28–0.54		382–570		130–465	379–401	51.4–53.7	134–168	215–275	
Apigenin	nd–9.49			1.10–2.66								
Apigenin der †			0.23–0.37		382–570	1130–1310	130–465	379–401	30.5–49.8	101.9–128.7	164–208	6.79–17.31
Apiin			0.04–0.05									
Diosmin der									nd–1.31	nd–2.76	3.94–4.81	
Genistein	nd–1.78			1.43–2.00								nd–13.26
Genistein der									nd–20.88	nd–66.12	nd–103.21	nd–4.95
Hesperidin			nd–0.13									
Kaempferol	nd–2.75											
Myricetin	4.2–25.9			11.2–21.2								
Naringenin der									nd–2.58	nd–2.27	1.73–4.11	
Quercetin	nd–2.61			0.64–1.44								
**Total phenolic acids**		150	3.50–4.32			1440–3240		40.0–46.2	53.1–120.6	82.6–107.5	32.1–85.9	
Caffeic	151–766			443–766				0.09–0.58				
*p*-coumaric	nd–6.44			2.47–6.55				0.11–0.18				
*p*-coumaric der						1440–3240						
*p*-hydroxybenzoic	2.26–28.49		0.65–0.67					22.8–27.8				
*p*-hydroxybenzoic der				5.39–17.8								
*p*-aminobenzoic			0.71–0.77									
Ferulic	1.39–19.34		1.35–1.57	5.18–11.8								
Protocatechuic							nd–7.5	13.0–14.7				
Sinapic acid der.									53.1–120.6	82.6–107.5	32.1–85.9	
Vanillic der			0.61–0.94									
**Other phenolics**		530	4.1–4.2									
**Total free phenols**		1140	7.88–8.96		382–570		130–465	419–447	109–172	216–276	247–361	6.79–31.27

nd: not detected, i.e., below the detection limit; † der, derivative.

Therefore, almost all the data available in the literature refer to the soluble free fraction (Table 3, Table 4, Table 5 and Table 6), more abundant and with better bioaccessibility [94]. In *L. albus* seed samples, varying quantities of the flavonoids apigenin, apigenin derivative (der), catechin der, genistein, genistein der, kaempferol, myricetin and quercetin were detected, as well as the phenolic acids caffeic, cinnamic der, *p*-coumaric, *p*-coumaric der, *p*-hydroxybenzoic, *p*-hydroxybenzoic der, 2,4-hydroxybenzoic der, chlorogenic, vanillic, ferulic and protocatechuic [21,86,87,89,90,92,93,95]. In *L. luteus*, the flavonoids detected were apigenin der, diosmin der, genistein, genistein der and naringenin der [21,92], along with the phenolic acids caffeic, *p*-coumaric, *p*-hydroxybenzoic, vanillic, ferulic and protocatechuic [92,93,95]. In *L. angustifolius* instead the flavonoids apigenin, apigenin der, genistein and genistein der were scored as well as the phenolic acids caffeic, cinnamic der, *p*-coumaric, *p*-hydroxybenzoic, *p*-hydroxybenzoic der, chlorogenic, vanillic, ferulic, sinapic and protocatechuic [21,92,93,95,96,97]. Finally, in *L. mutabilis* the flavonoids apigenin der, catechin der, diosmin der, genistein and genistein der were recorded as well as naringenin der along with the phenylethanoids tyrosol and tyrosol der and the phenolic acids cinnamic der, *p*-hydroxybenzoic der, 2,4-hydroxybenzoic der and vanillic der [44,93]. The most abundant phenolics were the flavonoids (85–100% of total phenolics), followed by the phenylethanoids (not detected–13%) and the phenolic acids (not detected–2%). Genistein der (464.69 mg/kg DM) and genistein (135.78 mg/kg DM) represented 67% and 20% of total flavonoids, respectively, corresponding to 64% and 19% of total phenolics [93].

**Table 4:** Free phenolics (mg/kg DM) of bitter (bit) and debittered (deb) seeds of *L. luteus*.

Reference	[87]	[88]	[21]	[89]	[90]	[91]
	bit	bit	bit	bit	bit	deb
	*n* = 1	*n* = 2	*n* = 2	*n* = 4	*n* = 2	*n* = 1
**Total flavonoids**	650	6.12–15.75	1380–1438	706–1144	1246–1508	399
Apigenin der †		nd–7.35	138–142	706–1144	1246–1508	27.82
Apiin		0.08–1.30				
Diosmin der						98.43
Genistein						40.39
Genistein der						230.00
Hesperidin		0.15–0.53				
Kaempferol		nd–0.17				
Luteolin		nd–1.14				
Luteolin der		nd–5.48				
Morin dihydrate		nd–4.67				
Naringenin der						2.03
Rutin		nd–0.40				
Rutin der		nd–0.60				
**Total phenylethanoids**						49.42
Tyrosol der						49.42
**Total phenolic acids**	60	6.29–6.39		10.0–27.6	41.5–82.1	
Anthranilic		nd–0.08				
Caffeic		nd–0.10			1.02–1.22	
*p*-coumaric		0.56–4.47			0.03–0.68	
*p*-hydroxybenzoic		0.54–4.55			1.06–2.24	
Ferulic		0.26–0.39				
Protocatechuic				10.0–27.6	35.9–73.6	
Gallic		0.18–0.33				
Vanillic		0.31–0.68				
**Other phenolics**	890	3.62–3.70				
**Total free phenols**	1600	16.03–25.84	1380–1438	719–1165	1288–1590	448.09

nd: not detected, i.e., below the detection limit; † der, derivative.

**Table 5:** Free phenolics (mg/kg DM) of bitter (bi), sweet (sw) and debittered (deb) seeds of *L. angustifolius*.

Reference	[68]	[88]	[21]	[91]	[92]	[96]	[93]
	bit/sw	bit	bit	bit	bit	bit	deb
	*n* = 55	*n* = 3	*n* = 2	*n* = 2	*n* = 2	*n* = 1	*n* = 1
**Total flavonoids**	1007–1643	1.84–2.91	784–1027	666–692	687–731	20.1	14.82
Apigenin			35.2–49.1				
Apigenin der †		0.17–0.25	735–992	666–692	687–731		14.82
Apiin		0.12–0.19					
Diosmin der							
Genistein						20.10	
Genistein der							
Hesperidin		0.19–0.29					
Luteolin		1.23–2.31					
Naringenin der		0.03–0.05					
Rutin der		nd–0.09					
**Total phenolic acids**	50.4–199.4	1.87–2.71			58.0–58.1	4.36	
Anthranilic		0.031–0.035					
Caffeic		nd–0.11			0.62–0.63		
Cinnamic der						0.30	
*p*-coumaric		0.46–0.62			0.34–0.42		
*p*-hydroxybenzoic					42.7–43.7	4.60	
*p*-hydroxybenzoic der							
Ferulic		1.10–1.47					
Protocatechuic					12.5–13.8		
Vanillic der		0.31–0.40					
**Other phenolics**		3.20–4.45					
**Total free phenols**		7.68–9.44	784–1027	666–692	745–789		14.82

nd: not detected, i.e., below the detection limit; † der, derivative.

The estimates of total free phenols may vary widely (see Table 3, Table 4, Table 5 and Table 6) due to the different genotypes tested, the diverse edaphic and climatic growing conditions, the varying testing methodologies, etc. Siger et al. [92] compared three different lupin species and noticed an increasing free phenolic content from *L. albus* (433 mg/kg DM) to *L. angustifolius* (767.0 mg/kg DM) to *L. luteus* (1439 mg/kg DM). A similar trend was reported by Czubinski et al. [21]: *L. albus* (476 mg/kg DM), *L. angustifolius* (906 mg/kg DM) and *L. luteus* (1409 mg/kg DM); by Magalhães et al. [90]: *L. albus* (223 mg/kg DM), *L. angustifolius* (679 mg/kg DM) and *L. luteus* (960 mg/kg DM); and by Ferchichi et al. [88]: *L. albus* (8.42 mg/kg DM), *L. angustifolius* (8.37 mg/kg DM) and *L. luteus* (20.93 mg/kg DM) Likewise, Estivi et al. [93] observed growing concentrations of total free phenolics in *L. albus* (9.23 mg/kg DM), *L. angustifolius* (14.82 mg/kg DM), *L. luteus* (448 mg/kg DM) and *L. mutabilis* (731 mg/kg DM). The diversity within and among species is probably due to genetic selection and adaptation to cropping areas with different soil, climate and pathogens [88].

The literature available on the phenolic composition and antioxidant capacity of lupins deals mainly with raw seeds (either bitter or sweet). The debittering process, characterised by a boiling step followed by repeated washings, leads to a drastic loss of water-soluble compounds [51,60], including, unfortunately, phenolics [44,57]. After debittering *L. mutabilis* seeds, Villacrés et al. [57] recorded a reduction in total phenolic content of up to 96–97%. Similarly, in the same species, Brandolini et al. [44] reported a 76% phenolic decrease, mainly due to phenylethanoid (−95%) and flavonoid (−71%) loss, while phenolic acids (−57%) were less affected. Estivi et al. [70] described that after debittering with 1.0% sodium chloride or 1.0% citric acid solution, the soluble-free fraction was still the most abundant and represented 51.8–67.0% of the total, while with water-debittering it could be as low as 35.1% due to longer treatment and greater wash-out.

**Table 6:** Free phenolics (mg/kg DM) of bitter (bit) and debittered (deb) seeds of *L. mutabilis.*

Reference	[44]	[44]	[93]
	bit	deb	deb
	*n* = 3	*n* = 3	*n* = 33
**Total flavonoids**	2739–2871	771–917	295–1340
Apigenin der †	154–200	5.27–36.94	2.70–27.29
Catechin der	926–986		nd–36.49
Diosmin der	159–328	33–95	7.23–168.19
Genistein	11.88–12.78	99–125	38.03–352.12
Genistein der	1280–1375	550–723	219.57–1062.84
Naringenin der	108–138	5.94–7.42	nd–11.58
**Total phenylethanoids**	494–1148	7.70–28.39	
Tyrosol	398–1044	2.17–19.29	nd–49.21
Tyrosol der	96–138	5.09–9.09	nd–60.27
**Total phenolic acids**	64–71	24–37	
Cinnamic der	6.39–30.39	5.75–14.36	nd–11.92
*p*-hydroxybenzoic der	1.99–12.38	0.87–8.02	
2,4-hydroxybenzoic der	4.04–4.53	7.73–14.45	
Vanillic der	24–45	0.54–9.11	
**Total free phenols**	3298–4025	803–956	341–1393

nd: not detected, i.e., below the detection limit; † der, derivative.

**Table 7:** Conjugated and bound phenolics (mg/kg DM) of bitter (bit) and debittered (deb) seeds of *L. albus*, *L. luteus*, *L. angustifolius* and *L. mutabilis*.

	Conjugated			Bound				
Reference	[70]	[70]	[70]	[44]	[44]	[70]	[70]	[70]
	deb *	deb **	deb ***	bit	deb *	deb *	deb **	deb ***
	*L. albus*	*L. albus*	*L. albus*	*L. mutabilis*	*L. mutabilis*	*L. albus*	*L. albus*	*L. albus*
	*n* = 2	*n* = 2	*n* = 2	*n* = 3	*n* = 3	*n* = 2	*n* = 2	*n* = 2
**Total flavonoids**	78.99–82.08	70.26–85.28	118.72–118.94	82–109	106–122	82.69–115.34	101.19–124.4	59.23–110.17
Apigenin der †	nd–39.49	nd–0.52	nd	10.20–13.13	12.26–14.68	0.15–1.77	0.42–0.84	1.35–6.01
Catechin der	nd–0.41	nd–0.88	3.54–4.06	19–31	14–17	23.94–26.24	27.37–28.25	8.74–11.7
Genistein	nd–2.68	nd–3.15	1.55–4.17		9.83–14.22	nd–2.13	nd–1.75	nd–1.77
Genistein der	33.63–73.04	63.22–82.8	105.62–111.76	40–59	60–76	40.41–78.23	53.71–83.44	29.23–83.91
Naringenin der	5.46–6.36	1.97–3.01	2.08–4.87	7.82–9.58	5.75–6.95	10.89–14.27	11.42–18.39	11.44–15.25
**Total phenolic acids**				108–145	54–72			
Cinnamic				2.57–3.43	6.53–7.99			
*p*-coumaric				0.08–0.16	0.01–0.23			
Ferulic				1.66–2.16	1.56–2.31			
*m*-hydroxybenzoic der				3.08–9.64	3.64–9.42			
*m*-hydroxybenzoic				3.71–5.24	6.10–11.05			
*p*-hydroxybenzoic der				55–70	17–23			
*p*-hydroxybenzoic				4.81–6.87	2.40–3.44			
Salicylic der				0.66–0.71	0.42–0.48			
Syringic der				19–31	5.72–7.28			
Vanillic				8.62–20.12	7.82–9.58			
**Total phenols**				190–254	161–187	83	101	59

nd: not detected, i.e., below the detection limit; † der, derivative; * debittered with H_2_O; ** debittered with 1%NaCl; *** debittered with 1% citric acid.

## 7. Conjugated and Bound Phenolics

As mentioned above, the available information on the conjugated and bound phenolics of lupins is scant. Estivi et al. [70], testing the effects of different debittering methods on phenolic compounds, observed that the soluble-conjugated and the insoluble-bound fractions represented 17.1–27.0% and 11.0–37.9% of the total, respectively. All the conjugated compounds detected belonged to the flavonoids (Table 7) and were apigenin der, catechin der, genistein, genistein der and naringenin der. Water debittering led to a lower conjugated phenolics content (79 mg/kg DM) than 1% saline solution (85 mg/kg) and, especially, 1% citric acid solution (119 mg/kg). Apigenin der and naringenin der were better preserved by the water method, and genistein der by the other two methods.

Brandolini et al. [44], studying the effect of processing in bitter seeds of *L. mutabilis*, found the flavonoids apigenin der, catechin der, genistein, genistein der (the most abundant flavonoid) and naringenin der in the bound fraction, as well as the phenolic acids cinnamic, *p*-coumaric, ferulic, *m*-hydroxybenzoic der, *p*-hydroxybenzoic der (the most abundant phenolic acid), salicylic der, syringic der and vanillic (Table 7). Debittering did not significantly alter the flavonoids (from 82–109 to 106–122 mg/kg DM), marginally decreasing the content of catechin der and naringenin der and slightly increasing those of apigenin, genistein and genistein der. On the other hand, the phenolic acids were halved (from 108–145 to 54–72 mg/kg DM), due to a strong reduction in syringic der and *p*-hydroxybenzoic der, *p*-hydroxybenzoic and vanillic acid, not balanced by a slight increase in *m*-hydroxybenzoic and cinnamic acids. Estivi et al. [70] found the same flavonoids (Table 7) in their debittered samples of *L. albus* and observed that the 1% NaCl solution better preserved them, followed by water and 1% citric acid solution (101, 83 and 59 mg/kg DM, respectively).

## 8. Influence of Technological Processes

Germination is an easy and useful process to increase the nutritional value of legumes and cereals; additionally, it causes a reduction in the content of alkaloids, phytynians, and oligosaccharides [98].

Rumiyati et al. [99] studied the effect of germination of Australian sweet lupin (ASL) seeds for nine days and noticed that the total phenolic contents and the antioxidant activity of germinated flour were significantly increased (700 and 1400%, respectively) following germination. Additionally, the concentration of phytosterols and the antioxidant activity of oil extracts from germinated ASL flour also increased significantly (300 and 800%, respectively). Similarly, Dueñas et al. [96] found that nine-day germination modified the quantitative and qualitative phenolic composition of lupin (*Lupinus angustifolius*) seeds with a significant increase in flavonoids (63% after nine days), total phenolic compounds (84%) and antioxidant activity. After germinating *L. albus* and *L. angustifolius* seeds for two weeks, Andor et al. [100] observed a significant increase in cinnamic acid derivatives (in particular, caffeic acid: 160% and 298%, respectively) and genistein (120% and 157%). These results are in close agreement with those of Fernández-Orozco et al. [101], who found an increase of 53% of total phenols in lupin sprouts after nine days, and Frías et al. [102], who observed an enhancement in the antioxidant activity of white lupin (*Lupinus albus*) after a nine-day germination and an increase in vitamins E and C content. 

Estivi et al. [69] germinated white lupin seeds for up to six days and observed that total carotenoid (mainly lutein) content increased up to 12-fold in comparison to ungerminated seeds. On the other hand, the total tocol content was almost unchanged; however, while before germination γ-tocopherol represented 95–99%, during germination, the α-tocopherol increased from not-quantifiable up to 74.8 mg/kg (37%), while the γ-tocopherol decreased progressively. Therefore, this shift in composition improves vitamin E activity.

The solid-state fermentation of *L. angustifolius* flour with different bacteria (*Bifidobacterium animalis* subsp. *lactis* DSM10140, *B. longum* subsp. *longum* DSM20097, *B. breve* DSM20213, *L. plantarum* DSM2648, *L. plantarum* KX881779 and *L. reuteri* KX88177), performed up to 72 h, always led to a significant increase in both total phenolic content (TPC) and antioxidant capacity [103,104]. Likewise, Łopusiewicz et al. [105] observed an increase in TPC (almost two-fold) and antioxidant capacity in lupin beverages fermented for 21 days with commercial kefir grains. Khan et al. [106] explored a combination of germination and fermentation of *L. angustifolius* seeds with a tempeh starter fungus (*Rhizopus* sp.) and found that a 12-h germination increased the TPC from 4.8 to 10.9 mg gallic acid equivalent/100 g DM, while fermentation of the 12-h germinated seeds further improved the TPC to 47.7 mg gallic acid equivalent/100 g DM; the antioxidant capacity improved accordingly.

Brandolini et al. [44] examined the effects of different processing techniques (extrusion and spray-drying) on the carotenoids, tocols and phenolic compounds (mainly free phenols) of debittered seeds of three Andean lupins. They observed that the extrusion did not modify the content of tocopherols, slightly increased that of phenolics and marginally reduced (14.5%) that of carotenoids, while spray-drying diminished tocopherols, carotenoids and phenolics (30.0, 35.4 and 48.4%), mainly because of processing conditions; the coating agent dilution effect was minimal.

As mentioned in the introduction, lupin flour can be used to improve the nutritional profile of food products. However, the effect of thermal treatment on phenolics and other antioxidant compounds during food manufacturing has been scarcely studied. Rumiyani et al. [107] observed that muffins baked at 190 °C for 25 min after the addition of 4% or 6% lupin flour had greater total phenolic content and antioxidant activity than their batters and attributed this result to both the release of phenolic compounds from the cellular structures and the formation of phenolic products from thermal degradation. On the other hand, Villarino et al. [108] found that carotenoid recovery in ASL-wheat bread was significantly inferior to the raw composite flours, in the order of 15% (zeaxanthin), 24% (lutein), 48% (β-carotene) and 71% (α-carotene), corresponding to a 62% loss in total carotenoids. During dough mixing in the presence of water and oxygen, lipoxygenases (LOX) oxidise the polyunsaturated fatty acids, causing the degradation of the antioxidant carotenoids. ASL has a high LOX capacity, and this may be the reason behind the low recovery rates of these carotenoids.

## 9. Conclusions

Lupins are a very promising alternative to animals as a protein source and have an outstanding content of antioxidant compounds. All four cropped species are good sources of proteins, dietary fibre and lipids, rich in unsaturated fatty acids, as well as of micro- and macro-elements, vitamins and antioxidants. Minor differences in antioxidant composition are reported among species. Carotenoids are more abundant in *L. angustifolius* and *L. albus*, tocopherols in *L. mutabilis* and *L. albus* and phenolics, mainly free flavonoids, in *L. mutabilis*; conjugated and bound phenolics are scarce and therefore are hardly studied.

The content of all these antioxidants is significantly increased by germination and fermentation. Technological treatments (e.g., debittering, extrusion, spray-drying and baking) may induce a partial loss of these compounds, which, however, are still present in relevant quantities in the end products.

Approaches to increase the availability of lupin antioxidants may include choosing the most promising accessions, adopting optimised debittering methods (not needed for the low-alkaloid varieties) that limit leaching of valuable compounds while also saving time and water consumption, germinating the seeds, selectively fermenting the flours and optimising the processing conditions. Major shortcomings in current knowledge are the limited information about the effect of processing on lupin antioxidants and the complete absence of data on their bioaccessibility, to be studied by appropriate digestion protocols on lupin-enriched ready-to-eat foods.

## Figures and Tables

**Table 1:** Carotenoid content (mg/kg DM) of bitter, sweet or debittered seeds of *L. albus*, *L. luteus*, *L. angustifolius* and *L. mutabilis*.

	N° Samples	(α + β)-Carotene	β-Cryptoxanthin	Lutein	Violaxanthin	Zeaxanthin	Total Carotenoids	Reference
***L. albus***								
sweet	*n* = 2			6.65–8.77	0.06–0.11	2.23–3.82	8.94–12.70	[31]
bitter	*n* = 3	0.39–0.82	0.01–0.02	0.85–3.74		0.65–1.02	1.90–5.26	[19]
debittered *	*n* = 2	2.25–7.86	0.18–1.58	7.91–9.33		1.86–2.30	12.20–21.07	[70]
debittered **	*n* = 2	2.44–6.89	0.11–1.21	8.74–9.35		1.89–1.95	13.23–19.34	[70]
debittered ***	*n* = 2	2.49–12.01	0.42–3.05	7.97–8.05		1.74–2.06	12.7–25.09	[70]
debittered	*n* = 3	0.39–0.89	0.03–0–06	0.84–5.07		0.34–1.17	1.60–7.14	[19]
***L. luteus***								
sweet	*n* = 2	0.18–0.25		4.30–4–50	0.04–0.06	1.31–1.81	6.12–6.33	[31]
bitter	*n* = 1	0.14	0.01	0.75		0.09	0.99	[19]
bitter	*n* = 1	0.12		3.02		0.99	4.13	[66]
debittered	*n* = 1	0.22	0.03	0.73		0.07	1.05	[19]
***L. angustifolius***								
sweet	*n* = 4	0.81–6.44		11.17–35.95	4.90–16.70	0.30–3.44	17.20–62.52	[31]
bitter/sweet	*n* = 55	3.6–7.7		13.8–30.1	2.1–10.8	5.6–15.6	30.6–56.5	[68]
bitter	*n* = 1	1.1	0.02	3.93		1.45	6.49	[19]
debittered		0.86	0.05	3.39		1.25	5.55	[19]
***L. mutabilis***								
bitter	*n* = 2	0.10–0.20		1.45–1.48		0.11–0.13	1.70–1.81	[69]
bitter	*n* = 3			1.06–1.39		0.09–0.11	1.15–1.50	[44]
bitter	*n* = 33	0.06–0.60	0.00–0.03	0.43–2.20		0.03–0.21	0.69–2.89	[19]
bitter	*n* = 1	0.26		3.3		0.71	4.51	[41]
debittered	*n* = 33	0.09–1.22	0.02–0.07	0.43–2.28		0.04–0.19	0.68–3.25	[19]
debittered	*n* = 3			1.04–1.33		0.11–0.12	1.15–1.45	[44]

* debittered with H_2_O; ** debittered with 1%NaCl; *** debittered with 1% citric acid.

**Table 2:** Tocopherol content (mg/kg DM) of bitter, sweet or debittered seeds of *L. albus*, *L. luteus*, *L. angustifolius* and *L. mutabilis*.

	N° Samples	α-Tocopherol	β-Tocopherol	γ-Tocopherol	δ-Tocopherol	Total Tocopherols	Reference
***L. albus***							
sweet	*n* = 2			126–130		126–130	[31]
bitter	*n* = 3	0.88–1.22	0.34–0.50	109–151	1.38–1.94	112–154	[19]
bitter/sweet	*n* = 8					121–131	[79]
	*n* = 6	0.92–1.65		61–130	1.05–2.55	63–134	[32]
bitter	*n* = 2	1.9–4.7		201–516	2.5–4.1	205–525	[78]
debittered	*n* = 3	1.45–1.76	0.29–0.99	131–218	2.08–2.44	136–223	[19]
debittered *	*n* = 2	0.26–0.45	1.13–1.36	192–234	1.99–2.09	195–241	[70]
debittered **	*n* = 2	0.50–0.56	0.52–0.85	176–214	1.61–2.26	179–218	[70]
debittered ***	*n* = 2	0.29–0.50	0.99–2.64	183–208	1.56–2.45	188–212	[70]
***L. luteus***							
sweet	*n* = 2			86–87		86–87	[31]
bitter	*n* = 1	0.83	1.07	187	1.26	191	[19]
bitter	*n* = 2	2.7–4.8		94–112	2.4–3.8	99–121	[78]
debittered	*n* = 1	0.5	0.89	257	2.26	261	[19]
***L. angustifolius***							
bitter							[69]
sweet	*n* = 4	2.67–5.16		61.24–91.52		65–97	[31]
bitter/sweet	*n* = 55	2.3–7.3		82.4–116.7		85–123	[68]
bitter	*n* = 1	2.08	0.24	89	0.67	92	[19]
debittered	*n* = 1	1.6	0.97	100.4	0.27	103	[19]
***L. mutabilis***							
bitter	*n* = 2	0.66–0.82	0.90–1.47	165.8–213.6	1.86–2.24	169–218	[69]
bitter	*n* = 33	0.27–1.24	0.70–3.77	169–245	0.43–3.36	172–250	[19]
bitter	*n* = 3	0.51–0.61	0.93–1.60	224–228	1.66–2.81	227–233	[44]
bitter	*n* = 1	2		171		173	[41]
	*n* = 7	1.42–5.51		69–90		74–95	[32]
debittered	*n* = 33	0.18–1.72	0.75–5.06	223–370	1.33–5.01	227–378	[19]
debittered	*n* = 3	0.63–0.72	1.55–2.72	275–363	2.37–3.02	280–379	[44]

* debittered with H_2_O; ** debittered with 1%NaCl; *** debittered with 1% citric acid.

## Data Availability

Data supporting the findings are available from the corresponding author upon reasonable request.
